# 

**Published:** 1892-05

**Authors:** 


### CONTENTS.

COMMUNICATIONS.

Discretion in the Performance of Dental Operations .... 203
On Edge .... 212
World's Fair Items .... 221

PROCEEDINGS.

Proceedings of the Mississippi Valley Dental Association .... 222

DENTAL LAW.

Amended Dental Law of Ohio .... 226

COMMENCEMENTS.

Philadelphia Dental College .... 230
United States Dental College .... 231
Baltimore College of Dental Surgery .... 232
Southern Medical College—Dental Department .... 233
Meharry Medical College—Dental Department .... 233
Missouri Dental College .... 234
Cincinnati College of Medicine and Surgery—Dental Department .... 234
Chicago College of Dental Surgery .... 235
Kansas City Dental College .... 236
University of Iowa—Dental Department .... 237
Royal College of Dental Surgeons of Ontario .... 237
Columbian University—Dental Department .... 238
Western Dental College of Kansas City .... 238
Tennessee Medical College—Dental Department .... 239
Pennsylvania College of Dental Surgery .... 239
University of Maryland—Dental Department .... 240
University of California—College of Dentistry .... 241
Indiana Dental College .... 242
Ohio College of Dental Surgery .... 242
New York College of Dental Surgery .... 243
Vanderbilt University—Dental Department .... 244
American College of Dental Surgery .... 245
Homœopathic Hospital College—Dental Department .... 246
Northwestern University—Dental Department .... 247

NOTICE OF DENTAL MEETINGS.

Dental Society of the State of New York .... 248
Florida State Dental Association .... 248
The American Dental Society of Europe .... 248
Illinois State Dental Society .... 249
American Medical Association—Section of Dental Surgery .... 249

EDITORIAL.

The Ohio Amended Law—Amended .... 250
A Remarkable Man .... 251
The Isaac Knapp Dental Coterie .... 252
Obituary—Dr. John Allen .... 253

